# *In vivo* endorectal dosimetry of prostate tomotherapy using dual MO*Skin* detectors

**DOI:** 10.1120/jacmp.v16i3.5113

**Published:** 2015-05-08

**Authors:** Sarah J. Alnaghy, Shrikant Deshpande, Dean L. Cutajar, Kemal Berk, Peter Metcalfe, Anatoly B. Rosenfeld

**Affiliations:** ^1^ Centre for Medical Radiation Physics University of Wollongong Wollongong New South Wales Australia; ^2^ Department of Medical Physics Liverpool and Macarthur Cancer Therapy Centre Sydney New South Wales Australia; ^3^ Ingham Institute for Applied Medical Research Sydney New South Wales Australia

**Keywords:** helical tomotherapy, MO*Skin* detector, Rectafix, rectal wall, prostate cancer

## Abstract

Verification of dose to the anterior rectal wall in helical tomotherapy to the prostate is important due to the close proximity of the rectal wall to the treatment field. The steep dose gradient makes these measurements challenging. A phantom‐based study was completed, aimed at developing a system for measurement of anterior rectal wall doses during hypofractionated prostate stereotactic body radiotherapy (SBRT) utilizing tomotherapy delivery. An array of four dual MO*Skin*™ dosimeters, spaced 1 cm apart, was placed on a replica Rectafix® immobilization spacer device. This Perspex probe is a more rigid alternative to rectal balloons, to improve geometric reproducibility. The doses at each point were measured in real time and compared to doses calculated by the treatment planning system (TPS). Additionally, distance‐to‐agreement (DTA) measurements were acquired to assist in the comparison of measured and predicted doses. All dual MO*Skin* detectors measured dose to within ±5% of the TPS at the anterior rectal wall. Whilst several points were outside of experimental error, the largest deviation from the TPS predicted dose represented a DTA of only 1.3 mm, within the acceptable DTA tolerance of 3 mm. Larger deviations of up to −11.9% were observed for the posterior and side walls; however, if acceptable DTA measurements are accounted for, then an agreement of 75% was observed. Although larger differences were observed at the other rectal wall locations, the overall effect of dose at these points was not as significant, given the lower doses. Despite the very high‐dose gradient region, real‐time measurements of the anterior rectal wall doses were within acceptable limits of TPS‐predicted doses. The differences between measured and planned data were due to difficulties in precisely locating each detector on the TPS dose grid, which presented large variations in dose between CT voxels in regions of steep dose gradients. The dual MO*Skin* system would, therefore, be a useful device for detecting errors in real time, such as patient shifts or incorrect setup, during tomotherapy of the prostate.

PACS numbers: 87.53.Ly, 87.55.km, 87.55.N‐

## I. INTRODUCTION

Hypofractionation may be advantageous in the treatment of prostate cancer due to the estimated low α/β ratio of prostate cancer.^(1)^ Stereotactic body radiotherapy (SBRT) can deliver this hypofractionated dose with examples of prescriptions varying from 35 Gy in 7 fractions to boosts of 19 Gy in 2 fractions. SBRT has been shown to produce low rates of acute low urinary and rectal toxicity (Grade II).^1^, ^2^, ^3^ Jabbari et al.^(2)^ completed a study with 20 patients treated with prostate SBRT as a monotherapy, with a prescription of 9.5 Gy in 4 fractions. Another 18 patients were given a boost with SBRT of 9.5 Gy in 2 fractions, after their treatment with external‐beam radiation therapy (EBRT) and androgen deprivation therapy (ADT). They found that 42% had acute Grade II gastrourinary and 11% of patients had acute Grade II gastrointestinal toxicity. There was no Grade III acute toxicity found; however, two patients experienced late Grade III gastrourinary toxicity.^(2)^

In 2010 in a study by Katz et al.,^(1)^ 73 patients were treated with 45 Gy EBRT along with an SBRT boost. The boost prescription varied between patient groups, and the various prescriptions were 18 Gy in 3 fractions, 19.5 Gy in 3 fractions, and 21 Gy in 3 fractions. It was found that less than 7% of patients experienced Grade II acute urinary and rectal toxicity. Only one patient experienced Grade III late toxicity.^(1)^ Oermann et al.^(3)^ in 2010 published a study using CyberKnife to deliver a SBRT boost of 19.5 Gy in 3 fractions along with an IMRT prescription of 50.4 Gy in 28 fractions to 24 patients, some also being treated with ADT. Thirteen percent of patients experienced Grade II gastrourinary toxicity and 4% of patients experienced Grade II gastrointestinal toxicity, with no Grade III observed.^(3)^

The proximity of the rectum to the prostate and the high radiation sensitivity of the rectal mucosa lead to negative aspects of this dose escalation technique. Doses higher than 70 Gy to the rectum (volume dependent) lead to a high incidence of rectal bleeding and other serious side effects (Grade II or higher) in the rectum, some of them chronic, which significantly reduce the quality of life for these patients. Various techniques to reduce the dose to the rectum have been tried previously, such as 3D conformal radiation therapy (3D CRT), intensity‐modulated radiation therapy (IMRT),^(4)^ and rectal balloons.^(5)^ These techniques have improved the situation to some extent, but rectal toxicity is still frequent, particularly when combined with high integral doses.

A system called Rectafix^(6)^ (AB Mimator, Uppsala, Sweden) has been developed to improve positioning and immobilization in the treatment of prostate radiation therapy.^7^, ^8^ This device increases the distance between the prostate and the rectum and, hence, only a very small volume of the rectal wall remains close to the prostate. This minimizes the volume of rectal mucosa that gets a high dose and, in this way, the rectal toxicity is reduced to very low levels. With growing interest in hypofractionated treatment to the prostate, dose escalation and sparing of the rectal mucosa is vital to realize the benefit of this hypofractionated treatment. Whilst the Rectafix is a good immobilization tool, the day‐to‐day variation in setup of a patient's treatment position (with the rectum in such a high‐dose gradient region), treatment delivery error and organ deformation (such as bladder filling), may still impact upon the dosimetry and dose to the rectal wall. Therefore, the monitoring of the dose in real time might address some of the issues and safety of the treatment. This is the motivation of this work.

Our study looks at building upon this technology and investigating its potential for a tomotherapy boost treatment of the prostate. A new replica ultrasound transducer has been developed at the Centre for Medical Radiation Physics (CMRP), University of Wollongong, for use in prostate tomotherapy. It is capable of *in vivo* measurements of absorbed dose to the rectal wall using dual MO*Skin* detectors positioned along the anterior edge of the probe. MO*Skin* detectors were chosen for this method due to their superior measurement capabilities of absorbed dose to the anterior rectal wall, such as their ability for measurements in high‐dose gradient areas.^5^, ^9^ The MO*Skin* (University of Wollongong, NSW, Australia), a MOSFET‐based dosimeter, is a silicon detector designed to measure doses at air/skin interfaces.^(5)^ These detectors have a reproducible water‐equivalent depth (WED) of 70 μm, which allows for measurements at this depth,^(5)^ consistent with ICRU recommendations for skin dosimetry.^10^, ^11^, ^12^ This is the depth that corresponds to the basal layer, which is the first radiosensitive layer of the epidermis.^9^, ^13^, ^14^ To achieve this depth measurement, a thin Kapton film (DuPont, Willmington, DE) overlays the gate, which acts as a buildup layer. The film also protects electronics from damage from, for example, moisture.^(9)^ The MO*Skin* detector has the advantage of a very small sensitive volume compared to other detectors, with its gate oxide thickness measuring 0.55 μm.^(9)^ The whole detector is 3 mm wide, 0.4 mm thick and, with the cable, 330 mm long,^(14)^ allowing for easy placement for *in vivo* dosimetry — for example, in cavities. This detector can permanently store accumulated dose and be read out without loss of dose information.^(15)^ A solid Perspex (Imperial Chemical Industries, London, UK) probe was designed containing four dual detectors. The probe can not only assist in immobilization, but can ensure the geometric conditions are the same for improved accuracy in plan delivery and dosimetric measurement in a high‐dose gradient region.

Consideration of angular dependence is important for using MO*Skin* detectors in tomotherapy due to the helical delivery of dose. MOSFET detectors have demonstrated an angular dependence in the past,^16^, ^17^ hence this needed to be determined for the current study. However, due to the fact that they cancel out the asymmetric shape of the MOSFET, dual MOSFETs were found previously to have an angular dependence of ±2.5% from all incident beam angles, and hence they can be used for treatments delivered by rotation such as tomotherapy.^(5)^ These dual MOSFETs (i.e., dual MO*Skin* detectors) were therefore utilized in this study.

The aim of this project was to determine whether dual MO*Skin* detectors could be arranged on a Perspex probe inserted into the rectum to provide real‐time absorbed dose measurements during a prostate boost treatment using helical tomotherapy. We hypothesized that MO*Skin* detectors could be used to indicate dose to the rectal wall within ±5% due to the fact that, in clinical use, there is normally a large percentage of dose points that fall outside of a 3% tolerance.^(18)^ The experiments were designed to benchmark detector accuracy.

## II. MATERIALS AND METHODS

### A. Construction of probe and treatment setup

**Figure 1:**
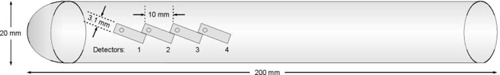
Solid Perspex probe with indentations on probe's surface to hold four MO*Skin* detectors. The small circles at the tip of each detector indicate the sensitive volume position.

A solid Perspex probe was designed and constructed. The probe was made with the dimensions of a BK Medical 8848 Ultrasound Transducer, which is the transducer used to guide catheter insertion in a prostate brachytherapy procedure, as well as being of the same dimensions of the Rectafix device. The Rectafix and replica probe were both composed of Perspex.^(7)^ The mass densities for the Rectafix and also the probe were $1.09-1.12\;g/{\text{cm}}^{3}$. The probe was combined with a one‐dimensional array of MO*Skin* detectors; therefore, four indentations were made on the probe's surface to position the detectors. Hence, the replica probe was utilized in place of the Rectafix since the probe could better position the detectors. The probe was 20 mm in diameter and 200 mm in length. The detectors needed to be positioned at an angle (shown in Fig. 1) so that the cables would not overlap the sensitive volumes, meaning that the cables had to wind around the probe. A study by Kwan et al.^(9)^ stated that the dimensions of the sensitive volume of the MO*Skin* are small enough so that its orientation relative to the gradient direction is not significant. The sensitive volumes are indicated by the small circles at the detector tips in Fig. 1. It can be seen in this figure that the dual detector positions will be referred to as 1, 2, 3, and 4, with 1 being placed most superior out of all dual detectors and 4 being the most inferior.

**Figure 2:**
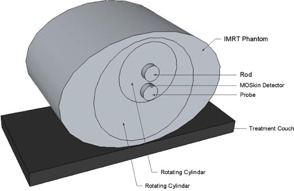
Experimental setup of IMRT phantom with MO*Skin* detector on probe.

**Figure 3:**
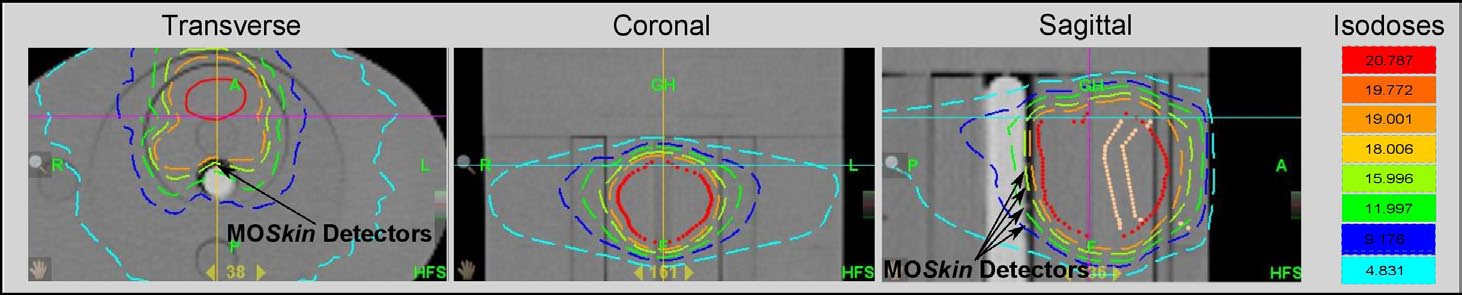
Treatment plan demonstrating position of MO*Skin* detectors in high‐dose gradient on surface of probe.

This method was tested using a TomoTherapy unit (Accuray, Sunnyvale, CA) at Liverpool Hospital, New South Wales, Australia. An IMRT Head and Torso Freepoint Phantom (CIRS model 002H9K; CIRS Inc., Norfolk, VA) was used for these measurements (Fig. 2). This phantom is constructed from tissue‐equivalent, epoxy materials with cylindrical cavities that allow rods to be inserted — for example, rods that can hold an ionization chamber or rods made of bone‐equivalent material. The cylindrical cavities can also be rotated to be placed in different positions.^(19)^ It is these cavities into which the probe can be inserted. The probe that was designed for this project had a diameter of only 20 mm; however, the phantom cavities have a diameter of 25 mm. Therefore, Perspex tubing with a wall thickness of 2.5 mm and length of 160 mm (equal to the length of the cavity plus excess to be able to remove the tubing easily) was used to create another layer around the probe so it could fit properly inside the phantom. The Perspex tubing was completely even around the probe; however, in Fig. 3, the gray scale changes on the CT image make it appear as though there is an air gap, due to the uncertainty in the CT image in detecting the edge of the cavity. There was no air gap present.

### B. Treatment planning

The CIRS phantom was scanned with a 35 cm field of view (FOV). These CT image datasets were imported as a phantom dataset into the tomotherapy treatment planning system (TPS). This was done to achieve the dose calculation grid required, since the calculation grid in tomotherapy planning depends upon the CT FOV utilized. For the phantom measurements, the delivery quality assurance (DQA) was created from the clinical plan with a Rectafix probe inserted into the phantom, scanned with a 35 cm FOV. All the clinical plans were optimized, as described in the Materials & Methods section D. The DQA plan was created by offsetting the phantom position to achieve the dose gradient at each detector as observed in the clinical plan (between the planning target volume (PTV) edge and rectal wall).

In the absence of the dose control system (DCS), which maintains a constant dose output to within ±2%, the output of the machine drops as a function of time.^20^, ^21^ The output of the machine drifts by more than 2% if the beam on time is longer than 7 min. Hence, to keep the machine output within 2%, the given fraction dose is split into three and delivered in three separate parts, with each part not exceeding 7 min in time length. This was done by adding 6 fractions instead of 2 fractions in the clinical plans. The tomotherapy boost was a hypofractionated schedule of 19 Gy in 2 fractions prescribed to the 90% isodose line, and this boost would be followed by IMRT with a prescription of approximately 46 Gy in 23 fractions. This prescription is currently being investigated by preliminary studies at Liverpool Hospital. A 9.5 Gy fraction is usually delivered by passing the patient through the TomoTherapy unit three times, with 3.17 Gy delivered per pass. Thus, in theory, the dose can be verified and corrected for after the first part, before the next part of the fraction is delivered. Correction may require another image‐guidance CT to ensure the patient has not moved, and/or, depending on the specific protocol of the hospital and the nature of the position change (for example, patient shift or organ deformation), a replan may be needed. Clinical trials, which are one of the suggested next steps of this study, would provide clearer answers as to what would offer the best option in this situation.

Image‐guidance CT scans were taken prior to treatment delivery to compare the detector position on the CT scan with the detector position on the preplan CT images to reduce positional deviations. Megavoltage (MV) CT scans using the TomoTherapy unit at approximately 3.5 MV^(22)^ were first taken of the CIRS phantom with the probe and MO*Skin* detectors inserted. However, on some of the images the MO*Skin* detectors were not clearly visualized, so kilovoltage (kV) CT datasets were obtained instead. The preplan CT scans were taken using a Siemens Sensation 4 Multislice CT scanner (Siemens Medical Solutions, Malvern, PA). Image guidance prior to treatment delivery on a patient is necessary as the probe would need to be inserted a minimum of twice for treatment.

Treatment plans were completed on the CT image datasets so that the TPS dose calculated at the positions of the detectors on the scan could be determined and later compared to the measured dose. The contours of volumes such as the PTV, clinical target volume (CTV), and critical organs/structures from a patient were overlaid onto the phantom CT images, so that realistic volumes could be utilized. A patient's treatment plan (from a real case) was adapted to the phantom's shape. The CTV was defined as the prostate, as well as extraprostatic disease, including extracapsular extension or seminal vesicle invasion. The PTV was a 5 mm extension to the CTV margin in all directions, except for the posterior margin where it was only 3 mm.

The prescription for a prostate treatment plan using helical tomotherapy in the clinic is 19 Gy in 2 fractions prescribed to the PTV. To achieve the sharp dose falloff, an inhomogeneous distribution is acceptable. Typically a prescription is 80%–90% of the target maximum dose to ensure the tumor boundary is on the steepest part of the dose gradient. This was achieved in this study by prescribing 19 Gy to the 90% isodose line, which covered 95% of the PTV.

The aim was to limit the rectal dose to 14–16 Gy, with the maximum occurring in the overlap region between rectum and PTV. Due to this overlap, where necessary, the PTV adjacent to the rectal volume may receive a minimum dose of 16 Gy. Otherwise a total dose of 19 Gy should be delivered to the PTV. The minimum dose to the CTV is 19 Gy and the maximum dose to the urethra is 21 Gy. These objectives were achieved for all plans used in this experiment. On each plan, the coordinates of each detector were determined, and the dose read out at these points. These readings were divided into 6 fractions and compared to the experimental results.

### C. Calibration of the MO*Skin* detector

The characterization of the MO*Skin* detector has been previously reported.^5^, ^23^ Calibration of the detectors was completed in this study. When dose was delivered to the MO*Skin* detectors, they were ‘immediately’ read out after irradiation using a clinical MOSFET semiconductor dosimetry system, which was also developed by CMRP. This device can measure the threshold voltages of up to five dual detectors simultaneously. A 30 sec time interval was invoked between irradiation and read‐out to allow the slight voltage increase, or ‘voltage creep‐up’,^(9)^ to plateau. This waiting period allowed the recombination of any charges that were not actually trapped.

The MO*Skin* detectors were calibrated using the TomoTherapy unit (approximately 6 MV^(22)^) using a static $5\times 40\;{\text{cm}}^{2}$ field at 85 cm source‐to‐surface distance (SSD). The detectors were placed at a depth of 1.5 cm in $30\times 30\;{\text{cm}}^{2}$ slabs of Gammex RMI solid water (Gammex Inc., Middleton, WI) and voltage readings acquired. The readings were compared to CC13 ionization chamber dose measurements that were also taken at 1.5 cm depth of solid water to determine the calibration factor. Readings from both MO*Skin* detectors and ionization chamber were acquired over a period of 1 min. Each MO*Skin* detector was calibrated facing both upwards and then downwards, so that an average calibration factor was determined for each detector orientation. An average calibration factor was then found between the upwards averaged calibration factor and downwards average calibration factor. The same detectors were used in treatment delivery.

**Figure 4:**
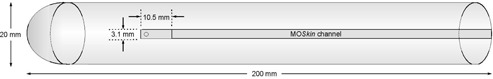
Solid Perspex probe with indentations on probe's surface to hold one MO*Skin* detector. The small circle at the tip of the detector indicates the sensitive volume position.

**Figure 5:**
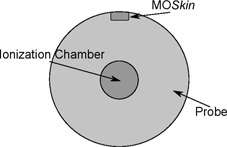
Cross section of Perspex probe demonstrating locations of single MO*Skin* detector and CC13 ionization chamber.

To test whether the MO*Skin* detectors were capable of measuring the dose that was planned, a single detector on the surface of a probe specifically designed to hold one detector (shown in Fig. 4), was placed in a region of low‐dose gradient, using one of the prostate treatment plans. The reason for this was to determine whether discrepancies between measured and planned doses were due to the actual detector, or the high‐dose gradient that the detectors would later be positioned in. The point chosen for this was the isocenter, which was in the middle of the PTV; hence, why only one MO*Skin* detector was used. This measurement was compared to the TPS dose, and it was also verified by an ionization chamber. However, the identical reference point for measurement with both ionization chamber and MO*Skin* could not be matched, due to the dimensions and physical location within the probe. The MO*Skin* was at the surface of the probe, whilst the ionization chamber was at the center (shown in Fig. 5). This meant that the dose agreement needed to be reported separately and compared to the TPS dose at two different locations. TPS uncertainty between voxels was minimal due the low‐dose gradient region; hence, reporting the dose at two different locations was acceptable.

### D. Treatment delivery

The absorbed dose measurements were then carried out using dual MO*Skin* detectors on the probe. Treatment plans were completed and optimized. A $1.37\times 1.37\times 1.00\;{\text{mm}}^{3}$ (i.e., 1 mm slice thickness) dose grid was utilized for calculation of the plan dose. The dose prescription was delivered as per the treatment plans. Measurements were taken at three different angles by rotating the probe to quantify the effect at different positions on the rectal wall. However, the focus was on the anterior rectal wall, as the overall effect of dose at the other locations was not as significant, given the lower expected doses. Letting $\theta =0^{\circ }$ be the most anterior point of the rectal wall, the detector readings were acquired by rotating the probe to $\theta =0^{\circ }$, 90°, and 180°. The measurements were repeated three times and the average taken, and these experimental results were compared to the TPS data. The uncertainty was determined by using Student's *t*‐test.

## III. RESULTS

### A. Calibration of the MO*Skin* detector

The average calibration factors were determined to be 2.57±0.06 mV/cGy for the upwards‐facing detectors, and 2.36±0.04 mV/cGy for the downwards‐facing detectors. An average calibration factor between the upwards and downwards values was therefore determined to be 2.47±0.07 mV/cGy, which was applied to all dual MO*Skin* detector measurements for this experiment.

The other measurement completed was to determine whether the MO*Skin* detectors performed well in a region of low‐dose gradient. Hence a single detector measured the dose in the center of the PTV. The difference between planned and measured doses, for both MO*Skin* detector and ionization chamber, was found to be <1%, demonstrating that possible discrepancies observed in the following experiment were not due to the inability of the MO*Skin* detectors, but rather the high‐dose gradient region.

### B. Treatment delivery

**Figure 6:**
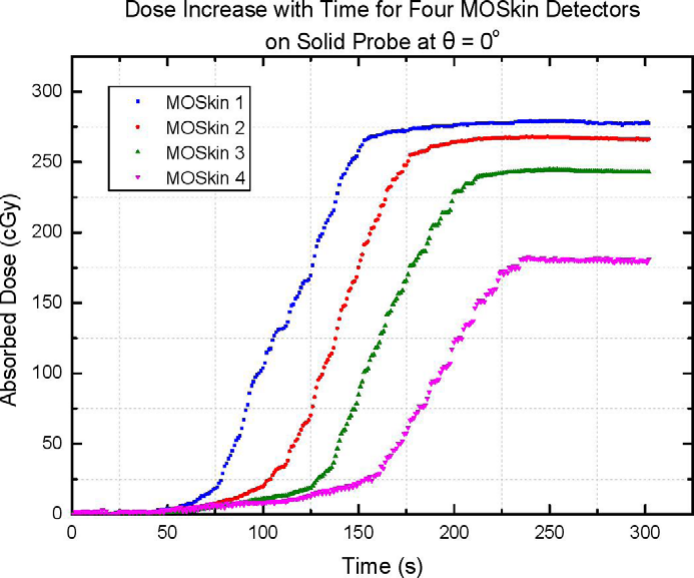
Graph of change in absorbed dose measured by each of the MO*Skin* detectors over time.

The planned dose prescription was delivered to the phantom and measured by the MO*Skin* detectors. One of the treatment plans can be seen in Fig. 3. Figure 6 is an example of the change in absorbed dose at each detector position over time, demonstrated by the readout of four single detectors. It can be seen that the dose increases at different times for each detector, depending on the relative position of each detector with respect to the radiation beam axis.

**Figure 7:**
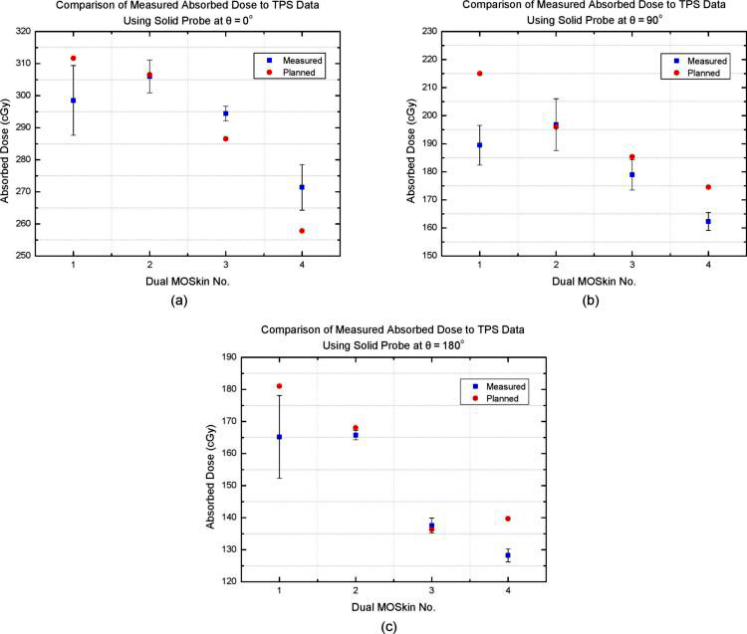
Graphs of absorbed dose taken from the TPS data in comparison to the dose measured experimentally, using four dual MO*Skin* detectors on a solid Perspex probe at (a) $\theta =0^{\circ }$, (b) $\theta =90^{\circ }$, and (c) $\theta =180^{\circ }$.

The recorded average changes in voltage were then converted to dose (D) per fraction (F) using the calibration factor. The errors in dose measurements were calculated using Student's *t*‐test, with sample size (N) equal to 3, two degrees of freedom, and *t*‐value equal to 4.303 for a 95% confidence level. The measured doses were compared to the planned doses and are displayed in Fig. 7. The average error was less than 3% of the measured doses. Dual detector 1 was believed to be slightly unstable, which was demonstrated by its larger error.

Distance‐to‐agreement (DTA) measurements were also completed. The DTA is defined as the distance between the location of the point of the measured dose to its corresponding isodose line.^(24)^ The acceptable DTA commonly used for photon beams is ≤ 3 mm.^25^, ^26^ Inability of the measured point to not only fall within the acceptable DTA distance but to fall outside the dose‐difference measurement indicates its failure to have produced a good measurement in a high‐dose gradient region.^18^, ^24^ Table 1 shows the DTA measurements.

**Table 1:** Table of distance‐to‐agreement (DTA) values.

*Angle* (θ)	*Detector*	*DTA (mm)*
0°	4	1.3
90°	1	4.3
	2	2.8
180°	1	3.9
	4	2.7

At $\theta =0^{\circ }$, dual detector 1 deviated by −4.2% from the TPS data, which was within tolerance. Dual detectors 2 and 3 also agreed with the TPS, deviating from the planned data by only −0.3% and 2.8%, respectively. The result from dual detector 4 differed from the TPS by 5.3%; however, its DTA measurement was 1.3 mm, which was within tolerance.

For $\theta =90^{\circ }$, dual detectors 2 and 3 measured results of 0.4% and −3.5% difference from the TPS, respectively. However, dual detector 1 varied by −11.9% from the planned doses and dual detector 4 differed by −7.0%. The DTA value for dual detector 1 was 4.3 mm, which was outside of tolerance. However, the DTA measurement for dual detector 4 was within tolerance at 2.8 mm.

For $\theta =180^{\circ }$, again, dual detector 2 and 3 showed good agreement of −1.3% and 0.9%, respectively. Dual detectors 1 and 4 measured doses of −8.7% and −8.2% variation from the TPS doses. The DTA measurement for dual detector 1 was outside the acceptable limit at 3.9 mm, however the DTA measurement for dual detector 4 was within tolerance at 2.7 mm.

## IV. DISCUSSION

For the anterior rectal wall, 75% of measurements agreed to within ±5% of the planned dose. However, detector 4 had a very small DTA measurement and, if experimental error is taken into account, its 5.3% deviation can be considered within the ±5% acceptance. Thus 100% agreement was observed at this location. The measurements taken at 90° and 180° were less significant than those measured at 0° due to the lower doses. The discrepancies at 90° and 180° can be explained by the greater difficulty in aligning the probe with the room lasers at these angles, causing a positional discrepancy. Uncertainty in the dose read‐out on the TPS can also explain these differences. The detectors on the preplan CT were only located at 0°; hence deviations at 90° and 180° would be subject to greater uncertainty than at 0° in determining the detector position on the TPS.

Between adjacent CT voxels on the treatment plan, a large difference in dose was observed. The point doses on the TPS corresponding to the measured doses varied from those CT voxels immediately adjacent by up to 29 cGy. By observing these steep dose gradients, it can be seen why such a small change in position can lead to a large change in dose, which may provide an explanation for variation between experimental results and the TPS. Further work in this study could be undertaken by simulating the clinical plans, thus reducing the discrepancies encountered when comparing measured and TPS data.

Any deviation that the more inferiorly placed detectors demonstrated may have been due to the slight anterior angling of the rectal volume on the plan compared to the phantom's rectal wall. As mentioned before, a patient's plan was adapted for the phantom plans so that the contours for the critical organs could be used. The contour of the rectum was curved, whilst the rectal wall was perfectly straight. The detectors were placed on the anterior side of the probe; hence, they may be angled away from their corresponding isodose line.

**Figure 8:**
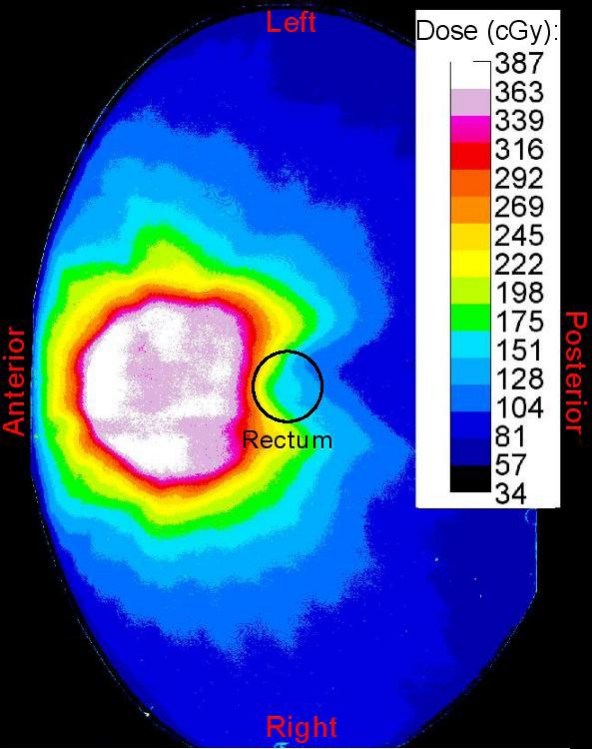
Dose map of tomotherapy plan.

Image artifacts were not thought to affect these results as there were no large perturbations in CT number around the probe position and no artifacts were visible by qualitative visualization of the CT images. The main source of uncertainty in this experiment was detector localization. It was difficult to place the detectors in the correct position, especially in a high‐dose gradient region. Figure 8 demonstrates the approximate position of the rectum in this high‐dose gradient region. The indents on the probe assisted in positioning the detectors; however, there would still be some discrepancy from the planned position. The MO*Skin* detectors could have also been affected by temperature or other physical factors, such as abrasion.

The results demonstrate that, even with a dose grid that is smaller than that utilized clinically for tomotherapy prostate boost treatments (at Liverpool Hospital), the reliance on positional accuracy in a high‐dose gradient region still has a significant effect on the results. The difference in dose across a $1.37\times 1.37\times 1.00\;{\text{mm}}^{3}$ voxel can still be quite large when compared to the size of the sensitive volume of a MO*Skin* detector, with its gate oxide thickness measuring 0.55 μm ^(9)^ and the thickness of the entire detector measuring only 0.4 mm^(14)^ — even when doubling this size as a dual MO*Skin* detector.

In terms of the patient tolerating discomfort from the probe, the fact that the treatment used in this experiment was a tomotherapy boost, to be used in conjunction with IMRT, means that the patient may only be required to have the probe inserted for the boost treatment. It is particularly important to verify the dose in the boost due the large fraction sizes (approximately 10.5 Gy per fraction), when compared to the smaller fraction sizes of the IMRT (approximately 1.8–2 Gy per fraction). The discomfit of the probe would therefore only need to be tolerated for 2 fractions instead of potentially 23 or more, making the treatment easier on the patient.

## V. CONCLUSIONS

Problems with prostate treatment using tomotherapy, such as prostate movement and the very steep dose gradient covering the anterior rectal wall, have led to uncertainty in dose to the rectal wall of the patient. A replica Rectafix probe was introduced to improve the dosimetry of prostate radiation therapy real time, specifically for tomotherapy. MO*Skin* detectors were chosen for this method due to their superior measurement capabilities of absorbed dose to the anterior rectal wall, such as ability for measurements in high‐dose gradient areas. This method was found to be successful, although there was still difficulty with positional reproducibility.

The next suggested step in this study would be to simulate the clinical plans before beginning placement *in vivo*. For tomotherapy, the patient is not normally treated with a probe inserted for standard fractionation. However, the improvement in positional reproducibility and added detectors will provide increased confidence in treatment delivery. The dual MO*Skin* system would be a good method for measurements of absorbed dose in tomotherapy prostate boost treatments, particularly for real‐time error detection, such as patient shifts or incorrect setup.

## ACKNOWLEDGMENTS

The author P. M. wishes to acknowledge financial assistance from the NSW Cancer Institute Clinical Leaders Program.
